# Transposon-mediated generation of *BCR-ABL1*-expressing transgenic cell lines for unbiased sensitivity testing of tyrosine kinase inhibitors

**DOI:** 10.18632/oncotarget.12943

**Published:** 2016-10-27

**Authors:** Konstantin Byrgazov, Chantal Blanche Lucini, Bettina Berkowitsch, Margit Koenig, Oskar A. Haas, Gregor Hoermann, Peter Valent, Thomas Lion

**Affiliations:** ^1^ Children's Cancer Research Institute, Vienna, Austria; ^2^ Department of Laboratory Medicine, Medical University of Vienna, Vienna, Austria; ^3^ Department of Internal Medicine I, Division of Hematology & Hemostaseology, Medical University of Vienna, Vienna, Austria; ^4^ Department of Pediatrics, Medical University of Vienna, Austria

**Keywords:** BCR-ABL1, tyrosine kinase inhibitors, Ba/F3, transposon-based gene transfer

## Abstract

Point mutations in the ABL1 kinase domain are an important mechanism of resistance to tyrosine kinase inhibitors (TKI) in *BCR-ABL1-*positive and, as recently shown, *BCR-ABL1-*like leukemias. The cell line Ba/F3 lentivirally transduced with mutant *BCR-ABL1* constructs is widely used for *in vitro* sensitivity testing and response prediction to tyrosine kinase inhibitors. The transposon-based *Sleeping Beauty* system presented offers several advantages over lentiviral transduction including the absence of biosafety issues, faster generation of transgenic cell lines, and greater efficacy in introducing large gene constructs. Nevertheless, both methods can mediate multiple insertions in the genome. Here we show that multiple *BCR-ABL1* insertions result in elevated IC_50_ levels for individual TKIs, thus overestimating the actual resistance of mutant subclones. We have therefore established flow-sorting-based fractionation of *BCR-ABL1*-transformed Ba/F3 cells facilitating efficient enrichment of cells carrying single-site insertions, as demonstrated by FISH-analysis. Fractions of unselected Ba/F3 cells not only showed a greater number of *BCR-ABL1* hybridization signals, but also revealed higher IC_50_ values for the TKIs tested. The data presented highlight the need to carefully select transfected cells by flow-sorting, and to control the insertion numbers by FISH and real-time PCR to permit unbiased *in vitro* testing of drug resistance.

## INTRODUCTION

**Figure 1:**
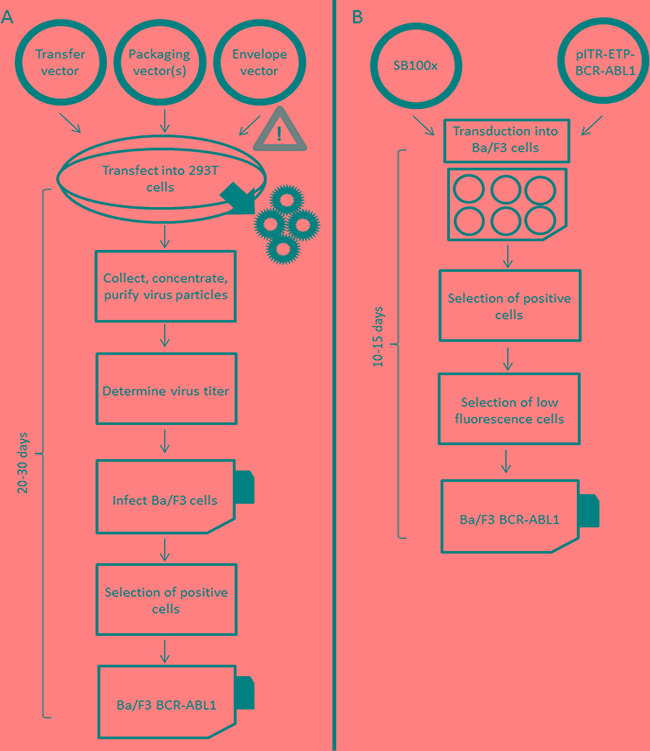
Workflows of lentiviral A. and Sleeping Beauty transposon-based B. transformation of Ba/F3 cells with *BCR-ABL1* fusion gene constructs

The Philadelphia (Ph) chromosome and the corresponding fusion gene *BCR-ABL1* are the genetic hallmarks of chronic myeloid leukemia (CML) and Ph-positive acute lymphoblastic leukemia (Ph+ ALL) [1]. The *BCR-ABL1* fusion gene [2], the alleged driver of the malignant phenotype in these leukemias, is known to activate several lines of downstream signaling including the MAP kinase, mTOR, JAK-STAT, and JAK-MYC pathways [3]. The introduction of imatinib and other TKIs has revolutionized the therapy of Ph+ neoplasia [4, 5], and shows great promise in the treatment of *BCR-ABL1*-like leukemias involving deregulated tyrosine kinases [6, 7]. In CML, most patients show good long-term responses to TKIs, but a considerable fraction eventually fail treatment and experience disease progression [8-11]. Clinical TKI-resistance is most frequently associated with mutations in the tyrosine kinase domain (TKD) of the *BCR-ABL1* fusion gene [12, 13]. The most commonly used model for *in vitro* sensitivity testing of novel TKIs and the prediction of resistance of emerging *BCR-ABL1* mutants [14-19] is the murine interleukin (IL)-3 dependent Ba/F3 cell line, which can be rendered IL-3 independent by lentiviral (LV) transduction with the *BCR-ABL1* tyrosine kinase [20]. This system has also been widely used as a model for the discovery and characterization of other oncogenic tyrosine kinases [21]. Nevertheless, LV-mediated introduction of oncogenic kinases into Ba/F3 cells has several relevant limitations and drawbacks. The transformation efficiency by LV transduction is rather low for large constructs due to the approximately 100-fold decreased success rate of RNA encapsidation into infectious particles for inserts over 6 kb in length [22], thus requiring laborious selection steps for the generation of transduced cell lines [23]. This problem also applies to full-length *BCR-ABL1* constructs which span over 5.8 kb. Moreover, lentiviruses tend to insert within transcriptionally active sites, thereby increasing the risk of mutagenesis and altered gene expression [24-27]. Insertion of multiple copies of the construct within the genome, which is commonly mediated by LV transduction [25], can also result in elevated expression levels of the transduced gene. Additionally, cells carrying more than one copy of a gene construct with oncogenic properties may have a growth advantage in culture, thus possibly affecting the readout of ensuing analyses. The observation of rather variable inhibitory concentration (IC_50_) values for individual TKIs reported for the same mutation in the BCR-ABL1 TKD might be attributable to this phenomenon [19, 28, 29]. Finally, the procedure of LV transduction can be very time consuming, and is associated with relevant biosafety issues. Based on these considerations, the *Sleeping Beauty* (SB) system, a synthetic DNA transposon designed to introduce defined DNA sequences into the chromosomes of vertebrate animals and humans, provides an attractive alternative for genetic transformation and insertional mutagenesis (Figure 1). The SB transposase targets TA-rich sites, preferentially the palindromic dinucleotide repeat ATATATATAT, in which the central TA is the canonical target site. In contrast to lentiviruses, there is no preference for coding or non-coding regions [30]. The SB system is a fast, simple and safe procedure for stable gene transfer facilitating efficient transfection even of large constructs [31-34]. However, similar to LV transduction, the SB system is also prone to generating multiple insertions in the genome which may be favored during the selection of engineered cells in culture [30, 35].

We have employed the SB system to establish Ba/F3 cell lines stably transfected with fluorescent proteins and *BCR-ABL1* constructs containing either wildtype (*BCR-ABL1*^WT^) or various mutants including G250E (*BCR-ABL1*^G250E^), E255V (*BCR-ABL1*^E255V^), T315I (*BCR-ABL1*^T315I^), F317L (*BCR-ABL1*^F317L^), and F359V (*BCR-ABL1*^F359V^). Lentivirally transduced Ba/F3 cell lines bearing the same BCR-ABL1 mutant constructs were tested for comparison. Fluorescence-activated cell sorting (FACS) with gating for low fluorescence was implemented to select cells displaying single gene construct insertions and homogenous transgene expression, and the efficacy of specific cell enrichment was documented by fluorescence in situ hybridization (FISH). The approach presented is applicable to cell line-based *in vitro* sensitivity testing of any mutation relevant in the clinical context, but was not designed for the monitoring of molecular remission or TKI resistance in patient samples. The data presented highlight the importance of using appropriately selected Ba/F3 cells for clinically relevant readout of TKI sensitivity testing *in vitro*.

## RESULTS

### Documentation of *BCR-ABL1* construct insertions by flow sorting and FISH analysis

**Figure 2:**
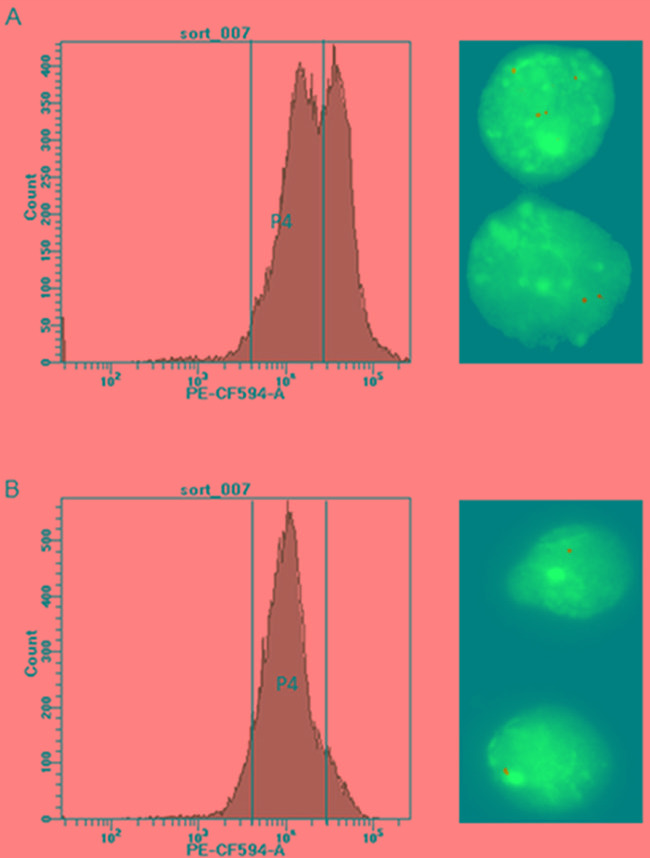
Flow-sorting of Ba/F3 cells **Panel A.** shows the histogram before sorting, with gate P4 encompassing a low-fluorescent cell fraction for targeted enrichment. Gate P4 was set above the auto-fluorescence level of Ba/F3 cells (4 × 10^3^ PE-CF594 -A fluorescence on the FACSAriaFusion instrument) and 2 × 10^4^ PE-CF594. On the right, the FISH image taken before flow-sorting documents the unsorted cells with two or more insertions (630x magnification). **Panel B.** shows the histogram of flow-sorted cells after one week in cell culture, with the great majority of cells located within gate P4. The FISH image on the right documents selective enrichment of cells carrying single insertions of *BCR-ABL1* constructs by gating for cells with appropriate fluorescence intensity.

**Figure 3:**
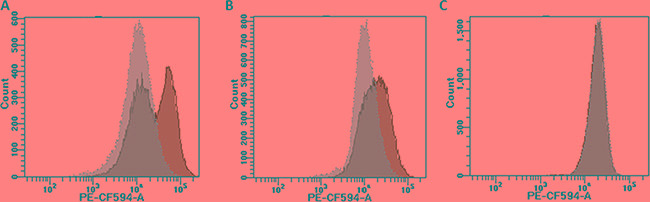
Different types of fluorescence distribution in unselected cells Unselected Ba/F3 cells carrying various BCR-ABL1 constructs displayed three types of the fluorescence distribution in flow-cytometry histograms: double peak **Panel A.** broad peak **Panel B.** and slender peak **Panel C.** The green histograms reflect unselected cells, and the changes after appropriate flow-sorting are shown in grey.

Ba/F3 cells carrying *BCR-ABL1*^WT^, *BCR-ABL1*^G250E^*, BCR-ABL1*^E255V^*, BCR-ABL1*^T315I^, *BCR-ABL1*^F317L/V^ or *BCR-ABL1*^F359V^ constructs were generated either by SB- or by LV-mediated gene transfer. After selection of successfully transformed cells using a fluorescence protein marker and IL-3 depletion, cells were either sorted for low fluorescence, as displayed in Figure 2A and Supplementary Figure S1, or remained unsorted. Both flow-sorted and unselected cell fractions were subjected to FISH analysis in order to assess the number of construct insertions in the genome. While 92-97% of cells obtained by low fluorescence-based flow-sorting showed single hybridization signals (Figure 2B, Supplementary Table S2), up to 67% of cells from the unselected fraction displayed two or more signals (Figure 2A, Supplementary Table S2). Flow cytometry-based analysis of the transformed cells displayed different types of fluorescence distribution (Figure 3). All LV-transduced Ba/F3 cells displayed a double peak, as presented in Figure 3A and Supplementary Figure S1, whereas SB-mediated transfection yielded three different types of peaks prior to selection. The analysis of unselected cells by FISH showed that the peak shape reflects the proportion of cells with multiple gene construct insertions (Supplementary Table S2). A double peak indicating a very high percentage of cells carrying an elevated number of insertions was observed in unselected Ba/F3 cells with *BCR-ABL1*^WT^ (Figure 3A). A broad peak resulting from a lower but still relevant number of cells with more than one insertion was detected in Ba/F3 cells with *BCR-ABL1*^T315I^ and *BCR-ABL1*^F317L^ (Figure 3B). Finally, unselected Ba/F3 cells with *BCR-ABL1*^G250E^*, BCR-ABL1*^E255V^ and *BCR-ABL1*^F359V^ revealed a slender peak revealing the presence of single construct insertions in the vast majority of cells (Figure 3C), indicating that further selection by flow-sorting for downstream analyses may not be required.

### *BCR-ABL1* expression levels in Ba/F3 cells with single and multiple construct insertions

**Figure 4:**
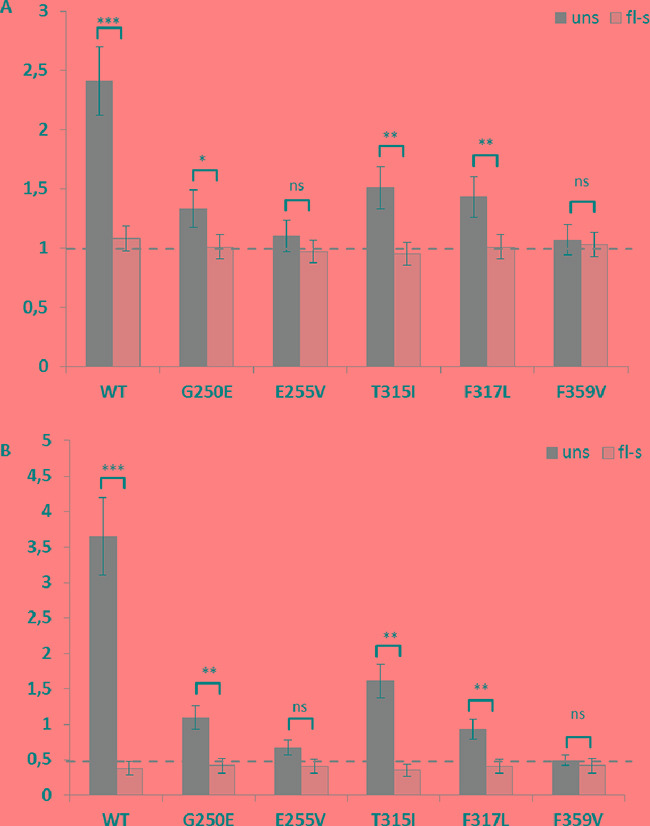
Real time PCR analysis of the copy number inserts and BCR-ABL1 expression analysis **Panel A.** shows the copy number of *BCR-ABL1* inserts in the diploid genome of Ba/F3 cells before (dark grey bars) and after (light grey bars) flow-sorting. **Panel B.** shows the relative expression levels of *BCR-ABL1* before (dark grey bars) and after (light grey bars) sorting. The dashed horizontal line highlights the average of one *BCR-ABL1* construct insertion in flow-sorted cells and the uniform *BCR-ABL1* mRNA expression level in flow-sorted cells uns, unselected; fl-s, flow-sorted; ** p<0.01; *** p<0.001; **** p<0.0001.

The increased copy number of *BCR-ABL1* insertions in unselected cell fractions displaying broad or double peaks in flow-cytometric analysis was also confirmed by quantitative PCR investigation of genomic DNA isolated from cells before flow-sorting. While most flow-sorted cells displayed a single copy of *BCR-ABL1* inserts per diploid genome, unselected cells revealed a significantly higher average number of *BCR-ABL1* insertions (Figure 4A and Supplementary Figure S2A). Real-time RT-PCR analysis indicated that flow-sorted cells carrying single insertions of the construct provide uniform mRNA expression levels in all transfected cell lines tested (Figure 4B and Supplementary Figure S2B). Moreover, *BCR-ABL1* expression in these cells corresponded to the levels detectable in clinical specimens of patients in chronic phase of CML, based on the experience of our European LeukemiaNet (ELN)-certified reference laboratory. By contrast, unselected Ba/F3 cells (with the exception of those revealing slender peaks upon flow-cytometry) revealed variable and significantly higher *BCR-ABL1* expression levels, apparently reflecting the predominant number of genomic insertions of the construct.

### Impact of the number of *BCR-ABL1* insertions on TKI responsiveness *in vitro*

Both unselected and flow-sorted Ba/F3 cells with wildtype or mutant *BCR-ABL1* constructs introduced either by SB-assisted transfection or LV-mediated transduction were subjected to *in vitro* survival assays with increasing concentrations of nilotinib, dasatinib, and ponatinib. The IC_50_ values obtained are displayed in Table 1 and Supplementary Table S3. Cells isolated by flow-sorting according to low fluorescence levels (Figure 2A) revealed IC_50_ values corresponding to the lowest measurements published previously [19, 36] while unselected cells displaying broad or double peaks at flow-cytometric analysis showed considerably higher values often indicating resistance to the TKI tested. In unselected *BCR-ABL1*^T315I^ LV-transduced Ba/F3 cells which displayed multiple insertions of the construct, as documented by FISH analysis (Supplementary Table S2), the IC_50_ determined was 35 nM (Supplementary Table S2), a value considerably higher than reported previously, and exceeding the effective plasma concentration of ponatinib achievable in the clinical setting with 45 mg per day [28]. Similar observations were also made in unselected Ba/F3 cells carrying other *BCR-ABL1* constructs and displaying broad or double peaks in FACS histograms, where the IC_50_ values correlated with the predominant number of insertions (Table 1) and (Supplementary Table S3).

**Table 1:** Analysis of *in vitro* response to different tyrosine kinase inhibitors

IC_50_ values of unselected and flow-sorted Ba/F3-SB-BCR-ABL1 (nM)			
BCR-ABL1 variant	Nilotinib	Dasatinib	Ponatinib
WT unselected	25.0 ± 6.0	4.2 ± 1.3	10.0 ± 2.0
WT flow-sorted	10.0 ± 3	2.3 ± 1.2	2.1 ± 0.8
*p-value*	*≤0.001*	*≤0.05*	*≤0.001*
G250E unselected	65.0 ± 15.0	10.0 ± 2.0	12.0 ± 2.0
G250E flow-sorted	50.0 ± 10.0	2.2 ± 0.5	5.7 ± 1.6
*p-value*	*≤0.01*	*≤0.001*	*≤0.001*
E255V unselected	1000.0 ± 150.0	11.0 ± 2.0	12.5 ± 1.5
E255V flow-sorted	900.0 ± 120.0	10.0 ± 1.5	10.5 ± 1.8
*p-value*	*≤0.1*	0.18	*≤0.1*
T315I unselected	9000 ± 1500	>10000	30.0 ± 5.0
T315I flow-sorted	7500 ± 1400	>10000	10.0 ± 2.0
*p-value*	*≤0.05*	*NA*	*≤0.001*
F359V unselected	67.0 ± 15.0	2.7 ± 1.2	6.5 ± 1.6
F359V flow-sorted	60.0 ± 12.0	2.5 ± 1.1	6.0 ± 1.3
*p-value*	0.22	0.67	0.41
F317L unselected	120.0 ± 20.0	20.0 ± 3.0	8.6 ± 2.7
F317L flow-sorted	54.0 ± 12.0	8.3 ± 2.6	2.5 ± 1.1
*p-value*	*≤0.001*	*≤0.001*	*≤0.001*

The indicated IC_50_ values were determined in unselected and low-fluorescent flow-sorted Ba/F3 cells carrying wildtype or different mutant *BCR-ABL1* constructs introduced by Sleeping Beauty (SB) transposon-mediated transfer. NA, not applicable.

### Kinase activity and proliferation of Ba/F3 cells with single and multiple insertions of *BCR-ABL1*

**Figure 5:**
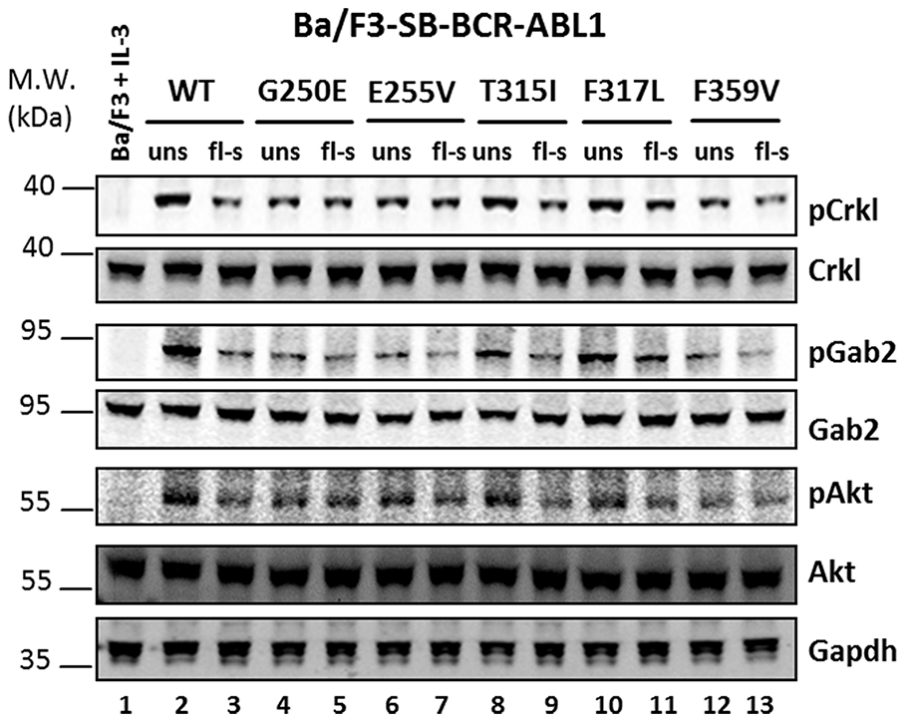
Western blot analysis of unselected and flow-sorted Ba/F3-*BCR-ABL1* cells Cell lysates were prepared from the parental Ba/F3 cells (growing in the presence of IL-3) (lane 1) and Ba/F3 cells bearing different *BCR-ABL1* constructs before and after flow-sorting, as indicated in Figure 2. The cell lysates were probed against different antibodies targeting the proteins indicated on the right. WT, wildtype; uns, unselected cells; fl-s, flow-sorted cells.

Western blot analysis was performed to compare the impact of single and multiple *BCR- ABL1* constructs in Ba/F3 cells on the ABL1 kinase activity and its downstream signaling by assessing phosphorylation of the target proteins Crkl, Gab2, and Akt [37, 38]. In comparison to flow-sorted cells displaying low-fluorescence (Figure 5, lanes 3, 9, and 11), unselected cells (Figure 5, lanes 2, 8, and 10) revealed elevated phosphorylation levels of the downstream proteins tested, unless the peaks of flow-cytometric analysis were slender (Figure 5, lanes 4, 5, 6, 7, 12, and 13). The unselected LV-transduced cells, all displaying a double peak, likewise reveal higher phosphorylation of the important downstream proteins in comparison to the flow-sorted cells (Supplementary Figure S3). The data indicate that the presence of two or more *BCR-ABL1* construct insertions in a high proportion of the cells investigated correlates with increased kinase activity.

**Figure 6:**
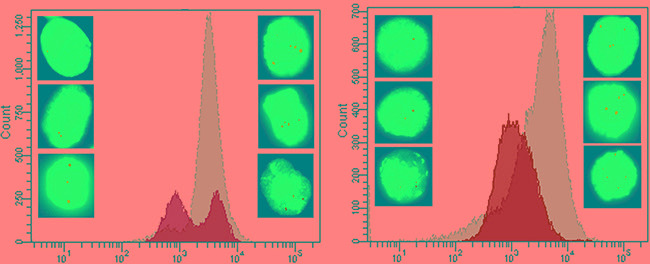
Outgrowth of cells with multiple *BCR-ABL1* construct insertions in culture **Panel A**. Unselected Ba/F3 cells SB-transfected with *BCR-ABL1*^T315I^ before and after expansion in cell culture for four weeks. Prior to culturing, the histogram of FACS-analysis revealed two peaks with different fluorescence intensities (green), and FISH-analysis indicated the presence of cells with 1-3 insertion sites of the *BCR-ABL1*^T315I^ construct (left side of the panel). After four weeks in culture, outgrowth of cells with higher fluorescence levels was evident (grey peak), and FISH-analysis showed predominance of cells with multiple insertion signals (right side of the panel). **Panel B**. Unselected Ba/F3 cells SB-transfected with *BCR-ABL1*^F317L^ revealing identical findings, i.e. outgrowth of cells with multiple insertions of the construct during cell culture.

In order to assess whether cells with multiple insertions have a proliferative advantage, unselected Ba/F3 cells with *BCR-ABL1*^T315I^ (Figure 6A) and *BCR-ABL1*^F317L^ (Figure 6B) constructs previously shown by FISH analysis to represent a mixture of populations with one, two and more insertions were investigated. The cells were grown in culture under standard conditions to study their proliferative behavior. After four weeks in culture, the cells were subjected to FACS analysis and the histograms revealed a major shift towards higher fluorescence levels. Subsequent assessment by FISH analysis showed multiple hybridization signals in virtually all cells analyzed (Supplementary Table S4) indicating the proliferative advantage of cells with several insertions of *BCR-ABL1* constructs.

## DISCUSSION

The importance of point mutations in the TKD of the *BCR-ABL1* fusion gene as a mechanism of TKI resistance has rendered *in vitro* sensitivity testing of emerging *BCR-ABL1* mutants a highly relevant diagnostic technique supporting clinical decisions [14-19, 29, 39]. The most commonly used approach to TKI sensitivity testing is based on exploitation of the murine Ba/F3 cell line carrying mutant *BCR-ABL1* constructs introduced by LV-mediated transduction [20]. Here we present an alternative technique, the transposon-based SB system, which offers advantages with regard to efficacy, speed, and safety [32-34, 40]. Nevertheless, we have demonstrated that both technical approaches can lead to multiple insertions of *BCR-ABL1* gene constructs which result in artificially elevated IC_50_ values for the TKIs tested. Reports indicating variable IC_50_ values for different TKIs tested against individual *BCR-ABL1* mutants might be attributable to this phenomenon. This is exemplified by published IC_50_ values for ponatinib against the P-loop mutation E255V including 16 nM [28], 33 nM [29], and even 56 nM [19]. The latter two values are beyond the achievable effective plasma concentration of the drug [28], and therefore indicate resistance. Such observations including the data presented underline the importance of generating Ba/F3 cells with single insertions of mutant *BCR-ABL1* constructs to provide a basis for adequate *in vitro* sensitivity testing of TKIs. Here we demonstrate that this goal is readily achievable by flow-sorting of SB-transfected Ba/F3 cells with gating for cell fractions with defined low fluorescence levels. The flow-sorting step does not seem to be required if the flow-cytometry histograms reveal a narrow peak indicating great predominance of cells with single insertions of the respective construct. While narrow peaks were observed in half of the cell preparations using the SB-transfection system, they did not occur in any of the preparations based on LV-transduction. Unselected preparations displaying broad or double peaks in flow-cytometry histograms were shown to contain relevant proportions of Ba/F3 cells with multiple *BCR-ABL1* construct insertions which resulted in artificially increased IC_50_ values for the TKIs tested. Moreover, the expansion of unselected *BCR-ABL1*-transformed Ba/F3 cells in culture was shown to favor proliferation and outgrowth of cells with multiple insertions, thereby preventing adequate *in vitro* sensitivity testing. The observation of greatly elevated IC_50_ values in these instances is in line with reports on amplification of the *BCR-ABL1* fusion gene as a relevant mechanism of resistance to kinase inhibitors [41-43]. Our data also revealed that multiple insertions correlate with enhanced kinase activity of BCR-ABL1 resulting in increased phosphorylation of the adaptor proteins Crkl and Gab2, which are prominent downstream targets implicated in leukemogenesis of Ph+ neoplasia [38]. Moreover, the mTOR signaling pathway was also upregulated in unselected cells, as demonstrated by elevated levels of phosphorylated Akt.

The generation of multiple gene construct insertions in Ba/F3 cells by LV- or transposon-mediated transfer has not been reported previously as a relevant problem in the context of *in vitro* drug sensitivity testing. Our data demonstrate that targeted flow-sorting of low-fluorescent *BCR-ABL1*-transformed Ba/F3 cells facilitates enrichment of virtually pure cell fractions carrying single insertions of the gene construct. Employment of this selection step is highly recommended for all cell preparations displaying broad or double peaks in flow-cytometry histograms to permit unbiased *in vitro* testing of drug resistance. The clinical relevance of our observations has far reaching consequences beyond Ph+ neoplasia. The rapidly increasing number of reports on *BCR-ABL1-*like leukemias involving a variety of activated kinases highlights the relevance of TKI-based treatment and *in vitro* prediction of sensitivity to these agents [6, 7, 44]. Appropriate modeling of activated and/or mutant kinases inserted at single sites into Ba/F3 cells, as presented in this report, is therefore of paramount importance for reliable prediction of treatment responses to kinase inhibitors *in vitro*, and opens improved possibilities for patient care in the growing field of precision medicine and targeted treatments.

## MATERIALS AND METHODS

### Cloning of *BCR-ABL1* into pITR-ETP SB vector

The full-length coding sequence of *BCR-ABL1* was amplified from cDNA of the K-562 cell line using the primers BCR_pITR_FW and ABL_pITR_RV (Supplementary Table S1) and cloned into *SfiI*-linearized pITR-ETP vector containing genes for puromycin resistance and the fluorescent protein tdTomato [33, 34, 40] using the In-Fusion Kit (Clontech Laboratories, Mountain View, CA, USA). The resulting plasmid pITR-ETP-BCR-ABL1^WT^ was subjected to site-directed mutagenesis using the complementary primer pairs G250E_s/G250E_as. E255V_s/E255V_as, T315I_s/T315I_as, and F317L_s/ F317_as (Supplementary Table S1) by implementing the QuikChange II XL Kit (Agilent Technologies, Santa Clara, CA, USA) according to the manufacturer's recommendations. Following targeted mutagenesis, the 4kb fragments including the *BCR-ABL1* TKD sequence between the SalI and SgrAI sites on each plasmid were sequenced and subcloned back into the pITR-ETP-BCR-ABL1^WT^ template to ensure that no passenger mutations have been introduced.

### Generation of *BCR-ABL1*-expressing Ba/F3 cells and selection by flow-sorting

The murine cell line Ba/F3 was obtained from DSMZ (Braunschweig, Germany, June, 2015) where the cells are authenticated using PCR-based methods. Ba/F3 were maintained in RPMI 1640 medium (Thermo Fisher Scientific, Waltham, MA, USA) containing 10% fetal bovine serum (Thermo Fisher Scientific), 1% penicillin-streptomycin (Thermo Fisher Scientific) and supplemented with 2 ng/ml murine IL-3 (Sigma-Aldrich, St. Louis, MO, USA). Cells were seeded at 3 × 10^5^ cells/ml and kept at a density below 1.5 × 10^6^ cells/ml. Nucleofection of pITR-ETP-BCR-ABL1 plasmids and transposase-encoding vector pcGlobin100x, using 4 μg and 1 μg, respectively, was performed using Cell Line Nucleofector® Kit V (Lonza, Basel, Switzerland) according to the manufacturer's instructions. Puromycin (Sigma-Aldrich) was added at 1 μg/ml to the medium 24 h after transfection, followed by selection for 2-5 days. All cells positive for the fluorescent protein tdTomato were isolated by flow-sorting using the FACSAriaFusion instrument (BD Bioscience, Franklin Lakes, NJ, USA) and grown in IL-3-containing medium to a density of 0.8-1 × 10^6^ cells /ml. IL-3-independent growth was assessed by washing the cells three times with IL-3-free medium followed by expansion in IL-3-free medium to 0.8-1 × 10^6^/ml. Finally, the cells were either left unsorted or enriched according to a defined low-fluorescence range (Figure 2A), and used for further experiments immediately after recovery. Fluorescence of sorted cells was controlled one week after sorting to assess the homogeneity of the cell population. For the unsorted *BCR-ABL1* mutant cells displaying T315I or F317L, the changes in fluorescence were measured after one month in culture. LV-transduced Ba/F3 cells [45] employed for control purposes were maintained in IL3-free medium and tested in parallel with SB-transfected cells in different experiments.

### Fluorescence in situ hybridization

The analysis by FISH was performed essentially as described [46]. Briefly, a digoxigenin-12-dUTP labelled pITR-ETP-BCR-ABL1 plasmid was used as hybridization probe to determine the number of *BCR-ABL1* construct insertions in the genome. Sheep antidigoxigenin-fluorescein isothiocyanate 1:100 (Roche Diagnostics, Basel, Switzerland) was used for immunodetection of hybridized probes. Signals were analyzed using a ZEISS Imager M2 fluorescence microscope (Carl Zeiss, Jena, Germany) and the Genesis software (Applied Spectral Imaging, Carlsbad, CA, USA).

### Tyrosine kinase inhibitor sensitivity assays

The TKIs nilotinib, dasatinib and ponatinib (all from Selleckchem, Houston, TX, USA) were obtained as 10 mM stocks in DMSO. Imatinib was not included because of its restricted efficacy in the presence of mutations. Serial TKI dilutions were prepared in phenol red-free RPMI 1640 containing 10% fetal bovine serum, and 1% penicillin-streptomycin, (Thermo Fisher Scientific) for subsequent in vitro sensitivity assays. *BCR-ABL1*-expressing Ba/F3 cells were seeded in 96-well plates at 3 × 10^5^ cells/ml and incubated with the respective TKI for 72 h at 37°C/5% CO_2_. Survival was assessed using the Vybrant^®^ MTT Cell Proliferation Assay Kit (Thermo Fisher Scientific) according to a protocol provided by the manufacturer. To estimate the IC_50_ of individual drugs for wildtype and mutant cells, smoothed dose-response curves were fitted using OriginPro 6.1 software (OriginLab, Northampton, MA, USA). The IC_50_ values were calculated by determining the mean of three independent experiments each performed in quadruplicates.

### Immunoblotting

A total of 15×10^6^ Ba/F3 cells SB-transfected or LV-transduced with *BCR-ABL1* were lysed using high-salt buffer (20 mM Tris*HCl, 400 mM NaCl, 0.5% NP40, 0.3% Triton X100) with Halt protease and phosphatase inhibitor cocktail (Thermo Fisher Scientific). The protein content was assessed using the Bio-Rad Protein Assay Kit II (Bio-Rad, Hercules, CA, USA). After denaturing the samples in NuPAGE^®^ LDS Sample Buffer (Thermo Fisher Scientific), proteins were separated by electrophoresis using NuPAGE^®^ 4-12% Bis-Tris Protein Gels (Thermo Fisher Scientific), transferred onto PVDF membranes (Thermo Fisher Scientific) with the XCell II™ Blot Module (Thermo Fisher Scientific), and treated with Odyssey Blocking Buffer TBS (LI-COR, Lincoln, NE, USA). Antibodies directed against the following targets were used to probe the membranes: phospho-Akt (Ser473), phospho-Gab2 (Tyr452), phospho-Crkl (Tyr207), Akt, Gab2, Crkl (all from Cell Signaling Technology, Danvers, MA, USA) and GAPDH (Abcam, Cambridge, UK). The DyLight™ conjugated antibodies (Cell Signaling Technology) were used for visualization of specific bands.

### Quantitative real-time PCR analysis

Genomic DNA was isolated using the DNeasy Blood and Tissue Kit (QIAGEN). Total RNA was isolated with the help of RNeasy Plus Mini Kit (QIAGEN) and reverse-transcribed by employing the High Capacity cDNA Synthesis Kit (Thermo Fisher Scientific). The number of *BCR-ABL1* double- or single-stranded DNA molecules was quantified on the Taqman 7500 Real Time PCR System (Thermo Fisher Scientific) using the primer probe set Hs03024784_ft (Thermo Fisher Scientific). Taqman mouse copy number reference assays Trfc and Tert (Thermo Fisher Scientific) were used for normalization of the number of *BCR-ABL1* inserts into the murine genome. The primer/probe set Mm01197698_m1 recognizing murine Gusb transcripts was used for normalization of the number of *BCR-ABL1* transcripts. All experiments were done twice in triplicates. The standard curves were built using the plasmids pITR-ETP-BCR-ABL1^WT^, pCMV6-Gusb (Origene) and mouse genomic DNA (Promega). Relative expression levels and copy number variations were calculated according to the ΔΔCt method.

### Statistical analysis

In the experiments described, two independent samples were analyzed in triplicates, unless indicated otherwise. Statistical analysis was performed using the OriginPro 6.1 software (OriginLab), and the two-sample independent T-test was employed for the calculation of significance values.

## SUPPLEMENTARY MATERIALS FIGURES AND TABLES


