# Supplementary material for: Transposon-mediated generation of BCR-ABL1-expressing transgenic cell lines for unbiased sensitivity testing of tyrosine kinase inhibitors
Source: Oncotarget. 2016 Oct 27;7(47):78083–94. doi: 10.18632/oncotarget.12943 (PMC5363645; doi:10.18632/oncotarget.12943)
Supplement: Supplementary file 1 [file oncotarget-07-78083-s001.pdf]

Transposon-mediated generation of *BCR-ABL1*-expressing transgenic cell lines for unbiased sensitivity testing of tyrosine kinase inhibitors

SUPPLEMENTARY FIGURES AND TABLES

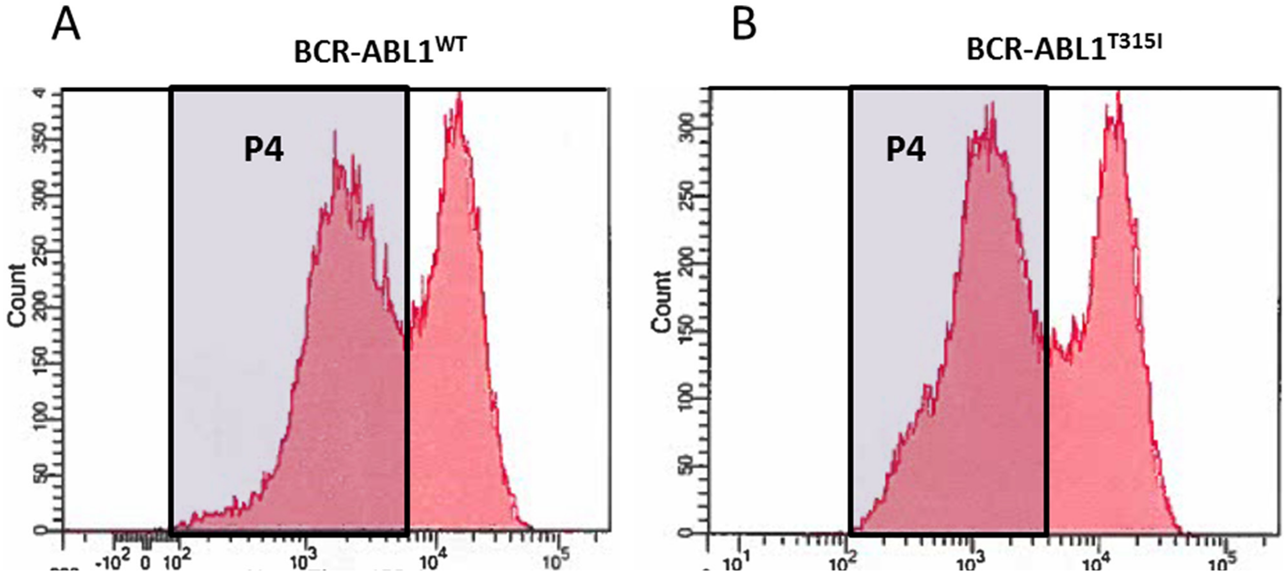

Supplementary Figure S1: Flow-sorting of LV-transduced Ba/F3-BCR-ABL1 cells. Histogram showing two representative examples of LV-transduced Ba/F3-BCR-ABL1 cells. Both cell lines displayed reveal double peaks reflecting various cell populations with multiple inserts, as documented by FISH (Supplementary Table S2). The gate for sorting was set as indicated by grey squares. Flow-sorting yielded cell populations with reduced *BCR-ABL1* genomic insertions, as documented by FISH and qPCR analyses.

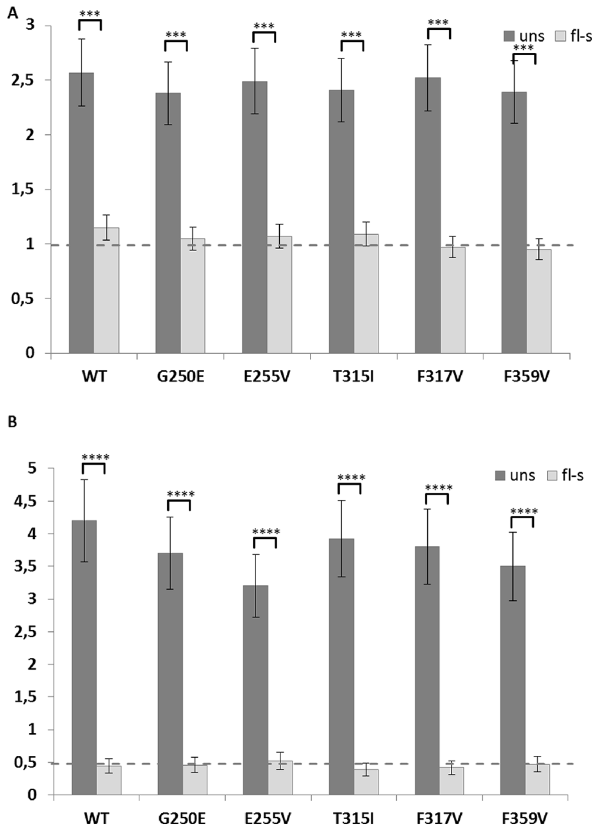

Supplementary Figure S2: Real-time PCR and BCR-ABL1 expression analysis. **Panel A.** shows the copy number of *BCR-ABL1* inserts in the diploid genome of LV-transduced Ba/F3 cells before (dark grey bars) and after (light grey bars) flow-sorting. **Panel B.** shows the relative expression levels of *BCR-ABL1* before (dark grey bars) and after (light grey bars) sorting. The dashed horizontal line highlights the average of one *BCR-ABL1* construct insertion per diploid genome in flow-sorted cells (Panel A), and the uniform *BCR-ABL1* mRNA expression level in flow-sorted cells (panel B). uns, unselected; fl-s, flow-sorted.

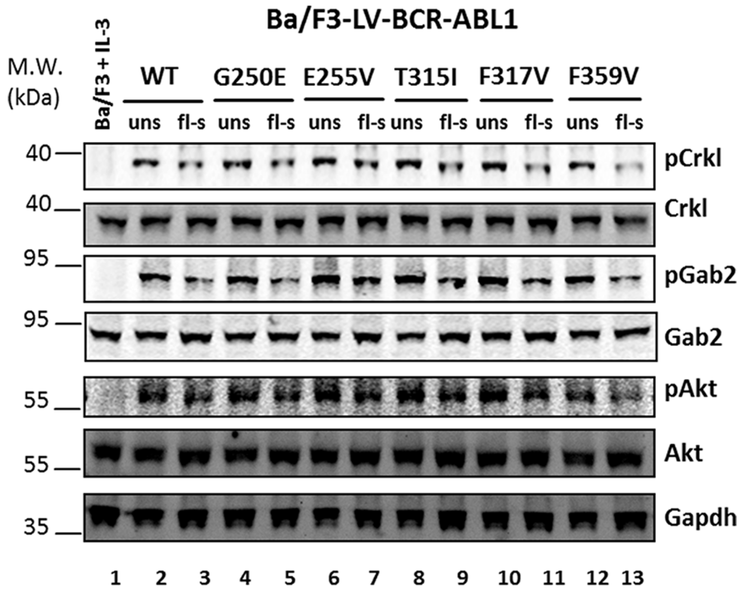

Supplementary Figure S3: Western blot analysis of unselected and flow-sorted LV-transduced Ba/F3-*BCR-ABL1* cells. Cell lysates were prepared from the parental Ba/F3 cells (growing in the presence of IL-3) (lane 1) and Ba/F3 cells bearing different *BCR-ABL1* constructs before and after flow-sorting, as indicated in Supplementary Figure S1. The cell lysates were probed against different antibodies targeting the proteins indicated on the right. WT, wildtype; uns, unselected cells; fl-s, flow-sorted cells.

Supplementary Table S1: Primer sequences employed in cloning

Primer designation	Sequence
BCR_pITR_FW	ACCCCAAGCTGGCCTATGGTGGACCCGGTGGC
ABL_pITR_RV	ATGGAAGCTTGGCCTCTACCTCTGCACTATGTCACTGATTTCCTT
G250E_s	CACAAGCTGGGCGAGGGCCAGTACGG
G250E_as	CCGTACTGGCCCTCGCCCAGCTTGTG
E255V_s	GCCAGTACGGGGTGGTGTACGAGGG
E255V_as	CCCTCGTACACCACCCCGTACTGGC
T315I_s	GCCCCCGTTCTATATCATCATTGAGTTCATGACC
T315I_as	GGTCATGAACTCAATGATGATATAGAACGGGGGC
F317L_s	CCCGTTCTATATCATCACTGAGTTAATGACCTACGGGAAC
F317L_as	GTTCCCGTAGGTCATTAACTCAGTGATGATATAGAACGGG
F359V_s	GATCTCTGTGGATGACGTTTTTCTTCTCCAGGTACTC
F359V_as	GAGTACCTGGAGAAGAAAAACGTCATCCACAGAGATC

Supplementary Table S2: FISH analysis of the transformed Ba/F3 cells

BCR-ABL1 construct	FISH signals unselected		FISH signals flow-sort	
	1	>1	1	>1
SB-WT	33%	67%	92%	8%
SB-T315I	92%	8%	96%	4%
SB-F317L	90%	10%	97%	3%
SB-F359V	95%	5%	96%	4%
LV-WT	40%	60%	97%	3%
LV-T315I	43%	57%	96%	4%
LV-F317V	42%	68%	96%	4%
LV-F359V	45%	55%	98%	2%

Number of genomic insertions of *BCR-ABL1* constructs documented by FISH signals in unselected and low-fluorescent flow-sorted Ba/F3 cells carrying wildtype (WT) or different mutant *BCR-ABL1* constructs introduced by the Sleeping Beauty transposon (SB)- or lentiviral (LV) -mediated transfer.

Supplementary Table S3: Analysis of in vitro response to different tyrosine kinase inhibitors

IC_{50} values of unselected and flow-sorted Ba/F3-LV-BCR-ABL1 (nM)			
**BCR-ABL1 variant**	**Nilotinib**	**Dasatinib**	**Ponatinib**
WT unselected	55.0 ± 13.0	3.7 ± 1.1	4.5 ± 1.5
WT flow-sorted	15.0 ± 4.0	0.7 ± 0.2	1.2 ± 0.2
p-value	*≤0.001*	*≤0.05*	*≤0.05*
G250E unselected	105.0 ± 15.0	12.0 ± 2.0	42.0 ± 8.0
G250E flow-sorted	45.0 ± 10.0	5.0 ± 1.0	8.5 ± 2.0
p-value	*≤0.001*	*≤0.001*	*≤0.05*
E255V unselected	2500.0 ± 200.0	25.0 ± 3.0	65.0 ± 14.0
E255V flow-sorted	940.0 ± 120.0	10.0 ± 1.0	18.0 ± 3.0
p-value	*≤0.001*	*≤0.001*	*≤0.001*
T315I unselected	9200 ± 1700	>10000	40.0 ± 8.0
T315I flow-sorted	8000 ± 1500	>10000	15.0 ± 3.5
p-value	*≤0.1*	*NA*	*≤0.001*
F359V unselected	150.0 ± 25.0	9.0 ± 2.0	24.0 ± 6.0
F359V flow-sorted	67.0 ± 12.0	3.5 ± 1.0	6.5 ± 1.5
p-value	*≤0.001*	*≤0.001*	*≤0.001*
F317V unselected	120.0 ± 30.0	250.0 ± 40	25.0 ± 6.0
F317V flow-sorted	35.0 ± 7.0	140.0 ± 20	12.0 ± 3.0
p-value	*≤0.001*	*≤0.001*	*≤0.001*

The indicated IC_{50} values were determined in unselected and low-fluorescent flow-sorted Ba/F3 cells carrying wildtype or different mutant *BCR-ABL1* constructs introduced by lentivirus(LV)-mediated transfer. NA, not applicable

Supplementary Table S4: FISH analysis of the unselected cells upon prolonged cultivation

	FISH signals unselected Week 1		FISH signals unselected Week 4	
**BCR-ABL1 construct**	**1**	**>1**	**1**	**>1**
SB-T315I	33%	67%	2%	98%
SB-F317L	90%	10%	25%	75%

Number of *BCR-ABL1* construct genomic insertions documented by FISH signals in unselected Ba/F3 cells upon cultivation for four weeks.
